# Comparative effectiveness of morning and evening aerobic exercise on weight loss and physical fitness in Chinese college students with overweight and obesity

**DOI:** 10.1186/s13102-025-01149-8

**Published:** 2025-04-28

**Authors:** Jingyu Sun, Nannan Jia, Yaning Xu, Dakai Guo, Tianfeng Lu, Jiajia Chen, Hang Chu, Zhangxiaohe Zhang, Ke Yao, Rongji Zhao, Tao Chen

**Affiliations:** Sports and Health Research Center, Department of Physical Education, Tongji University, Shanghai, China

**Keywords:** Morning or evening exercise, Body shape, Physical fitness, College students with overweight and obesity

## Abstract

**Background:**

Physiological and metabolic processes are influenced by biological rhythms. However, whether exercising in the morning or evening is more beneficial, given that these periods are two key time windows to incorporate exercise, has remained unclear. This study aimed to investigate the effects of morning versus evening exercise on weight loss, morphometric indicators, and physical fitness among Chinese college students with overweight and obesity.

**Methods:**

A total of 31 students with overweight and obesity (18–22 years old) were randomly assigned to exercise in the morning (7 am to 10 am, *n* = 16) or evening (6 pm to 9 pm, *n* = 15). The morning exercise group had an average age of 19.69 ± 1.01 years and a body mass index (BMI) of 27.13 ± 2.66 kg/m². The evening group had an average age of 19.47 ± 0.99 years and a BMI of 27.91 ± 3.40 kg/m². In the morning group, 87.5% of participants were male, while 73.3% were male in the evening group. The intervention was aerobic running exercise for 60 min, five times a week for 10 weeks. Measurements were taken before and after the 10-week intervention.

**Results:**

The BMI values of male and female college students were significantly and positively associated with vital capacity (*p* < 0.001) and significantly and negatively associated with vital capacity weight index, 50-meter, 800-/1,000-meter, standing long jump, and chin-ups and sit-ups test performances (*p* < 0.001). Participants who trained in the morning showed significant reductions in minimum waist circumference (MWC) (*p* = 0.043, d = 0.77), scapular skinfold thickness (ST) (*p* = 0.002, d = 1.30) and upper arm ST *(p* = 0.006, d = 1.05) compared with those who trained in the evening.

**Conclusions:**

Exercise in the morning, compared with that in the evening, has superior beneficial effects on weight loss and improving body shape in Chinese college students living with overweight and obesity.

**Trial registration:**

Chinese Clinical Trial Registry ChiCTR2400089594. Date of registration 11/09/2024.

**Supplementary Information:**

The online version contains supplementary material available at 10.1186/s13102-025-01149-8.

## Introduction

According to the latest statistics from World Health Organization (WHO), over 1 billion people worldwide suffer from obesity, including 890 million adults and 160 million adolescents and children [1]. Incidences of overweight and obesity among college students in China are increasing. In 2012, the prevalence of overweight (24 ≤ BMI ≤ 27.9 kg/m^2^) and obesity (BMI ≥ 28 kg/m^2^) was about 12.8% and 2.9% for male and female students, respectively [2]. In recent years, the overweight/obesity rate has increased to 20.8% and 8.4% for male and female university students, respectively [3, 4], an increase from 5.5 to 8%. Being overweight or obese significantly affects the physical health status of college students, especially in terms of speed, endurance, and explosive strength [5, 6]. College students are in a growth period or peak physical fitness, and poor lifestyle habits, such as overweight/obesity, developed in college tend to carry over into middle age, thereby leading to an increased risk of metabolic diseases in middle age and affecting lifelong health [7]. Accordingly, the physical health of college students, as the reserve talents for national construction, is a matter of national development.

Exercise has been consistently shown to offer substantial health benefits, including effective body weight management and enhanced physical fitness across all age groups [8, 9]. Studies have highlighted the distinct advantages of incorporating diverse exercise modalities, such as resistance training for muscle hypertrophy, flexibility exercises for joint mobility, and high-intensity interval training (HIIT) for metabolic efficiency, to optimize overall physiological adaptations [10–12]. Among the various forms of exercise, aerobic exercise has emerged as a key foundational intervention. ACSM’s Worldwide Survey of Fitness Trends for 2025 highlights the global popularity [13] and regional trends [14] in aerobic exercise. In particular, aerobic exercise has been identified as a safe and effective approach for improving various cardiovascular and metabolic risk factors in adults with obesity. These benefits include positive effects on body composition, lipid and glucose metabolism, blood pressure, and adipose tissue dysfunction [15, 16].

However, the optimal timing for aerobic exercise to maximize these metabolic benefits remains uncertain. Circadian rhythm-driven variations in energy metabolism and hormonal regulation may influence exercise-induced physiological adaptations at different times of the day [17]. As various physiological processes are influenced by biological rhythms throughout the day, responses to exercise may vary between morning and evening sessions. A few recent studies have attempted to examine the effects of exercise time of day (e.g., morning vs. evening) on weight loss [18–20] or physical fitness [21–23] to identify the optimal period for exercise. However, the existing evidence remains inconclusive owing to limited studies [24]. Thus, there are no specific recommendations on the optimal time of day for exercising to maximize health benefits.

The present study aimed to examine the correlations between body mass index (BMI) and physical fitness of college students and to comprehensively explore the similarities and differences in the health benefits of morning/evening exercise for Chinese college students with overweight and obesity by incorporating multidimensional indicators, such as body weight, morphometric indicators, and physical fitness. Findings of this study will offer new insights for promoting health among overweight and obese Chinese college students.

## Materials and methods

### Cross-sectional survey

College students were recruited to conduct a cross-sectional study. A total of 12,259 university students were included in the analysis. The physical fitness test indicators include height, weight, BMI, vital capacity, vital capacity weight index, 50-meter run, 800-/1,000-meter endurance run, sit-and-reach, standing long jump, chin-ups (male), and sit-ups (female) according to the Chinese Students’ Physical Fitness Test [25]. The test instruments are consistent with the national physical fitness test standards.

### Experimental intervention study

#### Participants

The sample size was calculated using data from a previous study (PMID: 28343364) [18], which indicated a large effect size (Cohen’s d = 0.84) for differences in weight loss between morning and evening exercise groups. To achieve 80% power (α = 0.05, two-tailed), a sample of 23 college students per group was needed. To mitigate attrition risk, we increased the target sample size to 60 college students. We successfully recruited 60 college students; however, 14 withdrew from the study prior to randomization. As a result, 36 college students were randomly grouped into either a morning exercise group (*n* = 18) or an evening exercise group (*n* = 18). Males and females were grouped together because differences in the results show no statistical significance for any of the variables measured. Those who satisfied the following criteria were qualified to participate in the study: (1) aged between 18 and 22 years, (2) BMI ≥ 24 kg/m^2^, (3) no diseases of the musculoskeletal or nervous system, and (4) willingness to participate in the test and sign an informed consent form. The subjects were asked to maintain a regular diet throughout the study. They were randomized into two groups: morning exercise group, which participated in morning exercise sessions (07:00–10:00); and evening exercise group, which participated in evening exercise sessions (18:00–21:00). Two and three individuals dropped out after being assigned to the morning exercise (*n* = 16) and evening exercise (*n* = 15) groups, respectively, resulting a final sample of 31 college students.

#### Experimental procedures

The participants completed the Basic Information Questionnaire (Supplementary Material 1), International Physical Activity Questionnaire Short Form [26], and Simplified Food Frequency Questionnaire [27] prior to the intervention to investigate basic information, their daily physical activity level, and their daily dietary energy intake. Body measurements, resting heart rate (RHR), blood pressure (BP), and physical fitness tests were administered to the subjects before and after the intervention. The exercise intervention was aerobic running. The intervention lasted 10 weeks, with a frequency of 5 days a week and 60 min a day. Exercise intensity was 60–75% of the maximum heart rate, as measured using a POLAR heart rate monitor. At the end of the 10-week intervention, the subjects were reassessed in accordance with the pre-intervention steps (post-test). All exercise and measurement procedures were conducted under professional guidance. The study was approved by the Ethics Committee of Tongji University (tjdxsr046).

#### Measurements

##### Anthropometrics and body composition

The participants’ height, waist, hip, and minimum waist circumferences (MWC) were measured using a standard tape, and waist-to-hip ratio (WHR) was calculated. Skinfold thickness (ST) was measured using a standard skinfold caliper (PZJ-01, Tixing, China). Weight, fat mass (FM), percentage of body fat (BF%), skeletal muscle mass (SMM), fat free mass (FFM), and visceral fat area (VFA) were calculated using the Body Impedance Analyzer (MC-980MA, TANITA body composition analyzer, Japan) and removal of metallic materials. BMI was calculated as the quotient of weight (kg) to height squared (m^2^). BMI categories were generally classified into underweight (< 18.5 kg/m^2^), normal (18.5–23.9 kg/m^2^), overweight (24–27.9 kg/m^2^), and obese (≥ 28 kg/m^2^) in accordance with the criteria of the Working Group on Obesity in China [28].

##### BP and RHR

All BP measurements were performed on the upper arm in a quiet room, with the subjects sat relaxed, using a calibrated and validated instrument (OMRON, model BP U724J) to obtain the systolic blood pressure (SBP), diastolic blood pressure (DBP), and RHR.

##### Physical fitness assessment

This study used eight fitness items to assess physical fitness, including vital capacity weight index (vital capacity adjusted by weight), speed (50-meter run), flexibility (sit-and-reach), cardiorespiratory endurance (800-/1000-meter endurance run for female and male, respectively), abdominal muscular endurance (sit-ups for female), upper body muscular endurance (chin-ups for male), and low body muscular endurance (1-min squat).

Vital capacity was measured using a calibrated spirometer (HK6800-FH, Hongkang Jiaye, China) and scored as the maximal expiratory volume of air (mL) after a maximal inhalation (measured twice). Thereafter, vital capacity weight index (mL/kg) was calculated as vital capacity (mL) divided by weight (kg).

The 50-meter sprint test required each participant to run as fast as they could for 50 m in a straight line on an athletic track. Speed was determined by the time taken to complete this test (test only once).

The sit-and-reach test required each participant to gradually reach forward with the fingers as far as they can, scoring the farthest point on the scale (the better record of two tests).

The 1-min squat test required the participants to complete as many cycles of squatting and standing up as possible in one minute, each time keeping the thighs at least parallel to the horizontal.

Male and female participants were asked to complete the chin-up/sit-up tests, respectively, as many times as possible in 60 s.

Male subjects were required to hold the overhead bar with both hands (palms facing away from the body), arms fully extended, and a chin-up was recorded when they used their arms to pull their body up until their chin was over the top bar. The next chin-ups required a starting position with extended arms.

Female subjects were instructed to perform sit-ups in a supine position with their knees bent, feet flat on an already secured floor mat, and hands on the back of their head. Thereafter, they were asked to raise their trunk until their elbows touched their thighs and return to the starting position.

In addition, male and female participants were required to complete the 1000- and 800-meter run tests, respectively. They were asked to run as fast as they could on the track. The time taken to complete this test was recorded.

#### Statistical analysis

Statistical analyses of all data were performed using the SPSS statistical package (IBM, SPSS Statistics 26). All continuous variables were expressed as means standard deviation (SD). Pearson correlation coefficients were calculated to examine associations between BMI and physical fitness variables in the cross-sectional study. Independent samples t-test was used to compare the baseline characteristics of the two groups, and to compare the changes in indicators before and after the intervention between two groups. To supplement key findings, effect sizes (Cohen’s d) were calculated for between group comparisons. To compare the within-group differences in indicators, namely, before and after the intervention, paired t-tests were performed. Confidence level for all statistical analyses was set at 95% (*p* < 0.05).

## Results

### Cross-sectional survey

#### Basic information of the physical fitness survey samples

A total of 12,259 college students who completed all physical fitness tests were enrolled in the final analysis, including 7,382 male students (60.22%) and 4,877 female students (39.78%), who were about 19 years old. The results of the height, weight, BMI, vital capacity, vital capacity weight index, 50-meter run, 800-/1,000-meter endurance run, sit-and-reach, standing long jump, chin-ups (male), and sit-ups (female) of the entire sample (i.e., male and female students) are shown in Table 1.

**Table 1:** Basic information of the physical fitness survey samples

	Total	Male	Female
*N* (*n*, %)	12,259 (100)	7382 (60.22)	4877 (39.78)
Age	19.38 ± 1.31	19.47 ± 1.34	19.25 ± 1.24
Height (cm)	170.25 ± 8.21	174.86 ± 6.12	163.26 ± 5.63
Weight (kg)	63.59 ± 12.05	68.49 ± 11.59	56.17 ± 8.39
BMI (kg/m^2^)	21.84 ± 3.24	22.37 ± 3.41	21.05 ± 2.78
Vital capacity (ml)	3633.76 ± 864.45	4109.05 ± 712.23	2914.34 ± 501.03
Vital capacity weight index (ml/kg)	57.54 ± 11.05	60.88 ± 10.81	52.49 ± 9.38
50-meter (s)	8.18 ± 1.08	7.51 ± 0.63	9.18 ± 0.81
800-/1,000-meter (s)	—	256.40 ± 41.30	244.15 ± 37.41
Sit-and-reach (cm)	18.05 ± 7.70	16.17 ± 7.96	20.89 ± 6.31
Standing long jump (cm)	211.45 ± 34.20	232.89 ± 23.09	178.98 ± 19.56
Chin-ups (num)	—	6.25 ± 5.27	—
Sit-ups (num)	—	—	36.90 ± 9.55

Note: *n* (%) indicates the number of people and their corresponding percentages, and the rest of the data were presented as mean ± standard deviation; 50-meter, 800-/1,000-meter, the longer the time, the slower the speed. Abbreviation: BMI, body mass index

#### College students’ BMI category statistics

The number and percentage of college students with different BMI categories are shown in Table 2. Fewer males than females were underweight or normal, and more males than females were overweight or obese (*p* < 0.001). In general, although 66.96% of college students had normal BMI, 16.53% and 4.84% were overweight and obese, respectively, with a combined overweight and obese rate of over 20% (21.37%). The overweight/obesity rates were 27.13% and 12.63% for male and female students, respectively.

**Table 2:** College students’ BMI category statistics

	Total	Male	Female	χ^2^	*p*
Underweight, *n* (%)	1431 (11.67)	733 (9.93)	698 (14.31)	387.388	< 0.001**
Normal, *n* (%)	8209 (66.96)	4646 (62.94)	3563 (73.06)		
Overweight, *n* (%)	2026 (16.53)	1526 (20.67)	500 (10.25)		
Obese, *n* (%)	593 (4.84)	477 (6.46)	116 (2.38)		

Note: *n* (%) indicates the number of persons and their corresponding percentages; ** indicates a highly significant sex-difference using Chi-squared test *p* < 0.01. Abbreviation: BMI, body mass index

#### Correlation between BMI and physical fitness test indicators of college students

Table 3 shows that the BMI values of the male and female groups were significantly and positively associated with vital capacity (*p* < 0.001). Moreover, the BMI values were significantly and negatively associated with each single physical fitness test performance (*p* < 0.001) in the male and female groups, except for sit-and-reach, which showed no significant correlation (*p* > 0.05).

**Table 3:** Correlation between BMI and physical fitness test indicators of college students

Indicators	Male BMI		Female BMI	
	*r*	*p*	*r*	*p*
Vital capacity (ml)	0.278	< 0.001**	0.198	< 0.001**
Vital capacity weight index (ml/kg)	-0.526	< 0.001**	-0.452	< 0.001**
50-meter (s)	0.238	< 0.001**	0.106	< 0.001**
800/1,000-meter (s)	0.248	< 0.001**	0.137	< 0.001**
Sit-and-reach (cm)	0.003	0.793	-0.007	0.614
Standing long jump (cm)	-0.260	< 0.001**	-0.129	< 0.001**
Chin-ups (num)	-0.348	< 0.001**	—	—
Sit-ups (num)	—	—	-0.079	< 0.001**

Note: ** indicates Pearson correlation test correlation significant *p* < 0.01

### Experimental intervention study

#### Basic characteristics of the participants

On the basis of the cross-sectional survey, an experimental intervention study was conducted with randomly selected college students who are overweight or obese. Similarities in their basic information were considered during grouping to ensure that no significant differences exist in various baseline parameters between the two groups (i.e., morning vs. evening exercise groups). The mean ages of the subjects in the morning and evening exercise groups were 19.69 ± 1.01 and 19.47 ± 0.99 years, respectively; and the mean BMIs were 27.13 ± 2.66 (kg/m^2^) and 27.91 ± 3.40 (kg/m^2^), respectively. The male/female ratios of the morning group were 87.5% and 12.5%, and those of the evening group were 73.3% and 26.7%. Table 4 shows the basic characteristics of the participants between the two groups, presenting no significant differences in their age, height, weight, BMI, physical activity, sitting time, and energy intake (*p* > 0.05).

**Table 4:** Characteristics of the participants

Variables	Morning Exercise Group (*n* = 16)	Evening Exercise Group (*n* = 15)	*p*
Age (year)	19.69 ± 1.01	19.47 ± 0.99	1.000
Height (cm)	176.69 ± 5.91	172.27 ± 8.51	0.102
Weight (kg)	84.53 ± 7.19	82.92 ± 12.54	0.168
BMI (kg/m^2^)	27.13 ± 2.66	27.91 ± 3.40	0.544
Physical activity (MET-min/w)	2217.94 ± 1792.45	2571.20 ± 2051.38	0.489
Sitting time (min)	482.50 ± 153.56	523.33 ± 139.37	0.445
Energy intake (Kcal)	1419.40 ± 744.49	1155.51 ± 465.65	0.916

Note: Data are presented as mean ± SD. Abbreviations: BMI, body mass index

#### Effects of exercise in the morning or evening on body morphology and composition of college students with overweight and obesity

After the intervention, the results of MWC (*p* = 0.043, d = 0.77), scapular ST (*p* = 0.002, d = 1.30), and upper arm ST (*p* = 0.006, d = 1.05) were significantly reduced in the morning exercise group compared with those in the evening exercise group. After the intervention, weight, BMI, MWC (*p* < 0.05), scapular ST, and upper arm ST (*p* < 0.01) were significantly decreased in the morning exercise group. However, the evening exercise group did not show such changes (*p* > 0.05) (Table 5).

**Table 5:** Comparison of body morphology and composition indicators of the morning and evening exercise groups in the pre- and post-tests

Indexes	Morning Exercise Group (*n* = 16)			Evening Exercise Group (*n* = 15)				
	Pre	Post	Change(95%CI)	Pre	Post	Change (95%CI)	*p*	d
Weight (kg)	84.53 ± 7.19	83.29 ± 7.00*	1.24 (0.02 to 2.45)	82.92 ± 12.54	82.69 ± 12.34	0.23 (− 0.49 to 0.95)	0.143	0.54
BMI (kg/m^2^)	27.13 ± 2.66	26.64 ± 2.31*	0.49 (0.05 to 0.94)	27.91 ± 3.40	27.85 ± 3.38	0.06 (− 0.19 to 0.31)	0.088	0.63
FM (kg)	23.13 ± 7.43	22.09 ± 6.35	1.04 (− 0.17 to 2.25)	25.21 ± 8.86	24.97 ± 8.79	0.25 (− 0.56 to 1.05)	0.262	0.41
BF%	27.16 ± 7.34	26.43 ± 6.85	0.74 (− 0.27 to 1.74)	30.23 ± 8.94	30.03 ± 8.73	0.21 (− 0.63 to 1.05)	0.398	0.31
SMM (kg)	58.17 ± 6.46	57.97 ± 6.55	0.20 (− 0.34 to 0.74)	54.61 ± 9.53	54.65 ± 9.37	−0.04 (− 0.54 to 0.46)	0.491	0.25
FFM (kg)	61.41 ± 6.66	61.21 ± 6.77	0.20 (− 0.37 to 0.77)	57.71 ± 9.89	57.73 ± 9.72	−0.02 (− 0.54 to 0.50)	0.549	0.22
VFA (cm^2^)	95.93 ± 19.09	91.74 ± 20.93	4.19 (− 1.41 to 9.78)	96.41 ± 31.86	94.78 ± 31.08	1.63 (− 2.06 to 5.31)	0.427	0.29
MWC (cm)	87.64 ± 5.44	86.12 ± 5.14*	1.52 (0.01 to 3.03)	87.17 ± 6.91	87.32 ± 6.90	−0.147 (− 0.79 to 0.49)	0.043	0.77
WHR	0.88 ± 0.05	0.88 ± 0.06	0.00 (− 0.02 to 0.02)	0.89 ± 0.05	0.89 ± 0.06	0.00 (− 0.03 to 0.03)	0.970	0.04
Scapular ST (mm)	37.56 ± 9.60	24.50 ± 7.74**	13.06 (10.03 to 16.10)	34.27 ± 10.63	33.77 ± 15.19	0.50 (− 6.36 to 7.36)	0.002	1.30
Upper arm ST (mm)	43.78 ± 6.48	29.53 ± 7.02**	14.25 (10.11 to 18.39)	42.77 ± 6.91	38.40 ± 12.31	4.37 (− 1.58 to 10.32)	0.006	1.05

Note: Data are presented as mean ± SD. * represents statistically significant difference within-group comparisons. P-value indicates differences in changes of variables between the morning and evening groups

Abbreviations: BMI, body mass index; FM, fat mass; BF%, body fat percentage; SMM, skeletal muscle mass; FFM, fat free mass; VFA, visceral fat area; MWC, minimum waist circumference; WHR, waist-to-hip ratio; ST, skinfold thickness

#### Effects of exercise in the morning or evening on RHR and BP of college students with overweight and obesity

After the exercise intervention, SBP in the morning exercise group was significantly lower than that in the pre-intervention level (*p* < 0.05). However, the evening exercise group did not show such changes (*p* > 0.05). After the intervention, RHR was significantly lower than that in the pre-experimental levels in the morning and evening exercise groups (*p* < 0.05) (Table 6).

**Table 6:** Comparison of RHR and BP of the morning and evening exercise groups in the pre- and post-tests

Indexes	Morning Exercise Group (*n* = 16)			Evening Exercise Group (*n* = 15)				
	Pre	Post	Change(95%CI)	Pre	Post	Change (95%CI)	*p*	d
RHR (bmp)	75.13 ± 8.37	70.56 ± 8.87*	4.56 (0.65 to 8.48)	80.33 ± 6.52	74.47 ± 9.06*	5.87 (0.30 to 11.43)	0.682	0.15
SBP (mmHg)	125.56 ± 10.64	119.63 ± 8.85*	-5.94 (0.10 to 11.78)	121.93 ± 14.28	116.07 ± 12.34	5.87 (− 0.32 to 12.05)	0.986	0.01
DBP (mmHg)	75.31 ± 4.48	74.81 ± 6.77	0.50 (− 3.03 to 4.03)	76.27 ± 8.48	74.27 ± 8.35	2.00 (− 0.74 to 4.74)	0.483	0.26

Note: Data are presented as mean ± SD. * represents statistically significant difference in pre- and post-test. P-value indicates differences in changes of variables between the morning and evening group

Abbreviations: RHR, resting heart rate; SBP, systolic blood pressure; DBP, diastolic blood pressure

#### Effects of exercise in the morning or evening on the physical fitness of college students with overweight and obesity

After the exercise intervention, 800-/1,000-m time in the evening exercise group was significantly lower than that in the pre-intervention level (*p* < 0.05). However, the morning exercise group did not show such changes (*p* > 0.05). After the intervention, performances in sit-and-reach and 1-min squat were significantly better than those in the pre-experimental level in the morning and evening exercise groups (*p* < 0.05) (Table 7).

**Table 7:** Comparison of the physical fitness indicators of the morning and evening exercise groups in the pre- and post-tests

Indexes	Morning Exercise Group (*n* = 16)			Evening Exercise Group (*n* = 15)				
	Pre	Post	Change(95%CI)	Pre	Post	Change (95%CI)	*p*	d
Vital capacity (ml)	4162.81 ± 714.73	4166.38 ± 702.70	−3.56 (− 106.50 to 99.37)	3834.27 ± 930.37	3846.87 ± 914.20	−12.60 (− 125.98 to 100.78)	0.900	0.05
Vital capacity weight index (ml/kg)	49.25 ± 7.39	50.02 ± 7.55	−0.78 (− 2.19 to 0.63)	46.88 ± 12.10	47.00 ± 11.64	−0.12 (− 1.22 to 0.99)	0.440	0.29
50-meter (s)	7.54 ± 0.58	7.46 ± 0.54	0.08 (− 3.06 to 2.77)	8.05 ± 1.08	7.94 ± 1.13	0.11 (− 0.11 to 0.34)	0.800	0.07
800-/1,000- m (s)	256.63 ± 26.60	250.69 ± 27.32	5.94 (− 5.68 to 17.56)	263.07 ± 40.24	254.13 ± 36.93*	8.93 (0.74 to 17.13)	0.660	0.16
Sit-and-reach (cm)	14.80 ± 8.32	16.67 ± 8.16**	−1.87 (− 3.22 to − 0.51)	9.63 ± 7.06	11.76 ± 7.42*	−2.13 (− 3.96 to − 0.31)	0.803	0.09
Chin-ups (male) (num)	3.86 ± 7.08	4.00 ± 7.19	−0.14 (− 3.06 to 2.77)	5.00 ± 4.20	5.91 ± 5.39	−0.91 (− 2.94 to 1.12)	0.368	0.19
Sit-ups (female) (num)	36.5 ± 10.61	39.5 ± 4.95	−3.00 (− 53.83 to 47.83)	35.25 ± 10.81	36.25 ± 9.18	−1.00 (− 7.63 to 5.63)	0.641	0.40
1-min squat (num/min)	49.75 ± 5.52	55.94 ± 6.85**	−6.19 (− 8.60 to − 3.78)	50.60 ± 8.71	55.20 ± 9.50*	−4.60 (− 8.27 to − 0.93)	0.439	0.28

Note: Data are presented as mean ± SD. * represents statistically significant difference in the pre- and post-tests. P-value indicates differences in changes of variables between the morning and evening groups

## Discussion

In this study, 12,259 students were randomly surveyed, of which 21.37% were overweight or obese. The prevalence of overweight/obesity was 27.13% in male students and 12.63% in female students. In addition, the distribution of the number of boys and girls with different BMIs was different, with a lower percentage of underweight and normal weight in boys than in girls, and a higher percentage of overweight and obesity in boys than in girls (Table 2). These findings are similar to those of others that being overweight or obese are more prevalent among college male students than female students [5]. Vital capacity can monitor human health and predict the risk of chronic respiratory diseases [29]. This study showed that college students’ BMI was positively correlated with vital capacity and negatively correlated with vital capacity weight index (Table 3), which is consistent with the results of previous studies [6]. Overweight or obese college students may have poor respiratory function, which was hypothesized to be related to the thickening of the chest wall fat and the reduction of lung volume caused by overweight or obesity. Meanwhile, they were poorer than underweight or normal weight college students in such qualities as speed, endurance, strength, explosive power, and flexibility (Table 3). This result is consistent with those of other studies [5, 6].

Recent research has shown that exercise performed at different times of the day have various effects on weight loss [20], body composition [20], cardiovascular function [30, 31], physical fitness [21–23, 32–34], and energy metabolism [35]. Studies indicate that morning exercise is more effective for weight loss in college students who are overweight and obese, with a significant reduction in waist circumference [18, 20]. In contrast, evening exercise does not produce similar changes. A 12-week study examining exercise timing found that morning exercise was particularly beneficial in reducing abdominal fat in healthy women [36]. Consistent with these findings, the results of the present study show that morning exercise is more effective in reducing waist circumference and skinfold thickness at the scapulae and upper arms in college students who are overweight and obese compared to evening exercise. Additionally, weight and BMI were significantly lower following morning exercise, with no significant changes observed after evening exercise (Table 5). Several factors may explain these findings. First, morning exercise has been shown to reduce post-exercise cravings for high-calorie foods more effectively than evening exercise, thereby contributing to greater weight loss benefits [37]. Second, metabolic rates are closely linked to the levels of acylcarnitine and branched-chain amino acids in skeletal muscle [38]. Studies have indicated that morning exercise, compared with evening exercise, is more effective in increasing the levels of acylcarnitine and branched-chain amino acids in skeletal muscle, thereby enhancing overall metabolic rate, further supporting weight loss effort [39]. Third, the expression of circadian genes (e.g., CLOCK and BMAL1) is influenced by the time of exercise and may further regulate energy metabolism [40]. This finding suggests that morning exercise may increase energy expenditure and improve fat metabolism by enhancing the circadian regulation of these genes. By contrast, evening exercise may be limited by circadian-regulated genes, resulting in less efficient fat oxidation. After overnight fasting, energy from carbohydrates, such as glycogen, is depleted, leading to a significant reliance on fatty acids for energy during morning exercise [41]. This shift may result in a substantial reduction in fat mass. Lastly, exercising in a fasted state, when insulin levels are considerably low and cortisol levels are markedly high, has been shown to promote fat oxidation [42]. Morning exercise may also enhance lipolysis by modulating such hormones as leptin and adiponectin [43]. Future studies could incorporate blood or urine samples to test subjects’ levels of key hormones and further explore the physiological mechanisms of morning and evening exercise in terms of body fat reduction.

Regular aerobic exercise has been shown to significantly lower resting heart rate and plays a crucial role in preventing cardiovascular disease [44]. However, no definitive conclusion has been reached on whether morning or evening exercise has a greater effect on blood pressure. Although this study showed that morning and evening exercises reduced resting heart rate with no significant between-group differences, the former significantly decreased systolic blood pressure (Table 6). These findings are consistent with previous research [45]. Moreover, blood pressure has been observed to be regulated by circadian rhythms, typically being higher in the early morning and lower at night [46]. Morning exercise may have a markedly pronounced effect on systolic blood pressure by reducing sympathetic activity and improving vascular endothelial function [47, 48]. However, some studies have reported opposing results, suggesting that mean arterial blood pressure is higher in healthy men after morning exercise compared with other times of the day [49]. This discrepancy may be caused by the rhythmic increase in blood pressure that occurs in the morning, which could mask the potential benefits of morning exercise on blood pressure regulation [46]. Therefore, the impact of exercise at different times of the day on blood pressure regulation and the underlying mechanisms warrant further investigation.

Endurance training at different times of the day has different effects [17]. Studies have shown that the body’s oxygen uptake and aerobic mechanical power output are higher with a 1000-meter cycling exercise training in the evening than with exercise training in the morning [50]. This finding has been confirmed by mouse models [51]. Consistently, the results of the current study also show that evening exercise significantly improved 800/1,000-m running performance, although there is no difference before and after morning exercise training (Table 7). In addition, studies have shown a significant negative correlation between BMI and flexibility qualities [52]. The current study show that morning and evening exercises improved sit-and-reach performance, but there is no between-group difference, which may be related to the fact that BMI decreased in both groups after the exercise intervention. However, there are no significant differences within and between groups for vital capacity, 50-m, chin-ups (male), and sit-ups (female), which may be related to the fact that the type of exercise used in this study was aerobic endurance running.

Other studies have demonstrated that combined aerobic and resistance training, HIIT, and resistance training significantly improve overall health, metabolic function, and cardiovascular health in individuals with type 2 diabetes, as well as those who are struggling with being overweight and obesity [53–55]. Molecular evidence further supports these findings. In particular, HIIT has been shown to enhance metabolic markers in obese individuals by modulating certain circulating adipokines [12]. Resistance training has also been found to influence the levels of specific myokines [56, 57], which play a crucial role in improving cardiovascular health. Future research could investigate the comparative effectiveness of morning versus evening sessions of resistance training, interval training, and combined aerobic and resistance training. This aspect could provide valuable insights into the optimal timing for exercise interventions aimed at improving metabolic and cardiovascular health.

Although this study offers valuable insights into the impact of exercise timing on physical health, several limitations should be acknowledged. First, despite prior power calculations and adjustments for attrition, the high dropout rate (*n* = 29) led to a reduced final sample size, thereby limiting statistical power to detect smaller between-group differences; therefore, findings should be validated in larger trials. Second, caution is warranted because this study did not monitor or control for potential confounding variables, including diet, sleep patterns, or physical activity beyond the prescribed intervention, all of which may have impacted the observed outcomes. Future research should aim to incorporate standardized assessments of these factors to enhance causal inference. Third, although this study found that morning exercise had a greater impact on improving body shape compared with evening exercise, it did not explore the interaction between exercise timing and different exercise modalities (e.g., resistance training vs. high-intensity interval training). Consequently, the potential synergistic effects of combining different exercise modalities remain unexplored. Previous studies have shown that HIIT and circuit resistance training (CRT) have more significant effects than moderate-intensity continuous training (MICT) on metabolism, cardiovascular risk factors, and body composition in obese men [58]. Future studies could explore whether or not exercise timing further affects the modality-specific benefits of different training approaches. Although previous studies have shown that aerobic exercise can impact bone health [59], bone health parameters were not addressed in this study. Therefore, future research could investigate whether or not morning and evening exercise have differential effects on bone metabolism. Lastly, this study focused exclusively on overweight and obese college students, thereby limiting the generalizability of the findings. Future research should aim to extend these findings to other age groups or populations with different metabolic profiles.

## Conclusions

This study explored the impact of different exercise timing on college students with overweight and obesity and found that morning exercise is more effective than evening exercise in improving the body morphology of these students. However, the effect of different exercise periods on physical function and physical fitness was not evident. To further identify the optimal exercise period for improving physical health, future research should refine the assessment of exercise timing. In addition, case studies could be used to explore the individual differences in how different exercise periods affect overweight and obese college students, providing a basis for developing personalized exercise prescriptions. Lastly, the mechanisms underlying the impact of exercise timing on health are complex. Hence, integrating sports medicine and molecular biology research could help uncover the fundamental principles, providing a scientific basis for the “time effect of exercise” and guiding future research directions.

## Electronic supplementary material

Below is the link to the electronic supplementary material.

Supplementary Material 1: Basic Information Questionnaire.

Supplementary Material 2: The study complies with the CONSORT guidelines and the CONSORT checklist is included as Supplementary Material 2.

## Data Availability

The datasets used and analyzed during the current study are available from the corresponding author on reasonable request.

## Abbreviations

BMI: Body mass index
RHR: Resting heart rate
BP: Blood pressure
MWC: Minimum waist circumference
WHR: Waist-to-hip ratio
ST: Skinfold thickness
FM: Fat mass
BF%: Percentage of body fat
SMM: Skeletal muscle mass
FFM: Fat free mass
VFA: Visceral fat area
SBP: Systolic blood pressure
DBP: Diastolic blood pressure
HIIT: High-intensity interval training
CRT: Circuit resistance training
MICT: Moderate-intensity continuous training
