# Supplementary material for: Comparative effectiveness of morning and evening aerobic exercise on weight loss and physical fitness in Chinese college students with overweight and obesity
Source: BMC Sports Sci Med Rehabil. 2025 Apr 28;17:101. doi: 10.1186/s13102-025-01149-8 (PMC12036172; doi:10.1186/s13102-025-01149-8)
Supplement: Supplementary file 1 — Supplementary Material 1: Basic Information Questionnaire. [file 13102_2025_1149_MOESM1_ESM.docx]

**Supplementary Material 1**

Basic Information Questionnaire

1. Did your doctor tell you that you are not suitable for physical exercise training? A. Yes B. No

2. Have you been diagnosed with any cardiovascular disease? A. Yes B. No

3. Have you been told by a doctor that your blood pressure is over 140/90 mmHg? A. Yes B. No

4. In the last 3 months, have you fallen or fainted while playing sports or doing physical exercise? A. Yes B. No

5. Do you like to eat high-calorie foods (foods high in carbohydrates such as starch and sugar, or animal offal and fried foods)? A. Yes B. No

6. In the last 3 months, have you smoked cigarettes (at least 1 cigarette per day)? A. Yes B. No

7. In the last 3 months, have you been drinking alcohol (at least once a week)? A. Yes B. No
